# CAFusion: A progressive ConvMixer network for context-aware infrared and visible image fusion

**DOI:** 10.1371/journal.pone.0339828

**Published:** 2026-01-08

**Authors:** Hafiz Tayyab Mustafa, Hamza Mustafa, Hassan Alhuzali, Mujtaba Asad, Zhonglong Zheng

**Affiliations:** 1 School of Computer Science and Technology, Zhejiang Normal University, Jinhua, Zhejiang, China; 2 Zhejiang Institute of Photoelectronics, Jinhua, Zhejiang, China; 3 Department of Electrical and Information Engineering, University of Cassino and Southern Lazio, Cassino, Italy; 4 Department of Computer Science and Artificial Intelligence, Umm Al-Qura University, Makkah, Saudi Arabia; 5 Institute of Image Processing and Pattern Recognition, Shanghai Jiao Tong University, Shanghai, China; Chuo University, JAPAN

## Abstract

Image fusion is a challenging task that aims to generate a composite image by combining information from diverse sources. While deep learning (DL) algorithms have achieved promising results, most rely on complex encoders or attention mechanisms, leading to high computational cost and potential information loss during one-step feature fusion. We introduce CAFusion, a DL framework for visible (VI) and infrared (IR) image fusion. In particular, we propose a context-aware ConvMixer block that uniquely integrates dilated convolutions for expanded receptive fields with depthwise separable convolutions for parameter efficiency. Unlike existing CNN or transformer-based modules, our block captures multi-scale contextual information without attention mechanisms, with computational efficiency. Additionally, we employ an attention-based intermodality multi-level progressive fusion strategy, ensuring an adaptive combination of multi-scale modality-specific features. A hierarchical multiscale decoder reconstructs the fused image by aggregating information across different levels, preserving low and high-level details. Comparative evaluations of benchmark datasets demonstrate that CAFusion outperforms recent transformer-based and SOTA DL-based approaches in fusion quality and computational efficiency. In particular, on the TNO benchmark dataset, CAFusion achieves a 0.769 score in the structural similarity index measure, a 2.07 percent increase as compared to the best competing method.

## 1 Introduction

Multisensor image fusion has emerged as a significant field of research, owing to its potential benefits and wide-ranging applications in various domains, including remote sensing, autonomous driving, medical diagnostics, and surveillance. Capturing detailed visual information has become easier with recent developments in imaging devices. However, single-modality imaging devices fail to capture scene details in complex environmental conditions such as low visibility. The combination of infrared (IR) and visible (VI) sensors, in particular, has proven to be beneficial for intelligent processing [1,2]. Under well-defined lighting conditions, VI sensors can capture fine details of the scene with high spatial resolution. IR sensors can identify the thermal radiation emitted by objects and display the structures of thermal objects that are resistant to changes in lighting. However, IR images typically lack fine features and have poor spatial resolution. Because of the visual differences between the various sensors, it is hard to combine the two types of images in a way that is visually appealing or to use them for higher-level vision tasks such as segmentation, detection [3], and tracking [4,5] to achieve the desired results. As a result, image fusion has been thoroughly studied as a potential substitute to combine the information of multiple sensors within a single image.

Over the past few decades, image fusion has been widely studied, and many attempts have been made to generate better-quality fused images. Researchers have developed several fusion approaches, which can be broadly divided into two categories: deep learning (DL)-based and traditional methods. Traditional techniques [6–9] mainly employ basic image processing concepts to VI and IR images and then combine them with specific fusion schemes to generate fused images. These approaches consist of the manual development of several components, such as image transformation from one domain to another with complicated fusion rules. As a result, most of these methods suffer from either loss of significant details or imbalanced information from both modalities into the fused output.

Recently, convolutional neural networks (CNNs) and their variants have gained popularity in image fusion applications, mainly because of their strong representation capabilities. Consequently, DL-based fusion methods overcome the limitations of manual design and outperform traditional techniques. The primary reason is that CNN can learn important image fusion parts in a single network. As image fusion is a generative task, therefore DL-based approaches mostly employ two baseline architectures: encoder-decoders [10–13] and generative adversarial networks [14,15]. However, these methods face significant challenges. The lack of ground truth images is one of the key challenges for DL models. Therefore, most DL-based frameworks adopt unsupervised learning to generate fused images. These methods often follow hierarchical architectures to extract local features. However, the global context information of the source images might be lost in reconstruction. Moreover, a one-layer fusion scheme for feature fusion also results in the loss of global and modality-specific information in the fused image. Recently, vision transformers and diffusion-based techniques [16–19] have been introduced to capture long-range dependencies of images. However, these approaches come with a critical drawback, such as significantly higher computational costs. This computational complexity limits their application in resource-limited environments, such as real-time systems or embedded devices.

**Fig 1:**
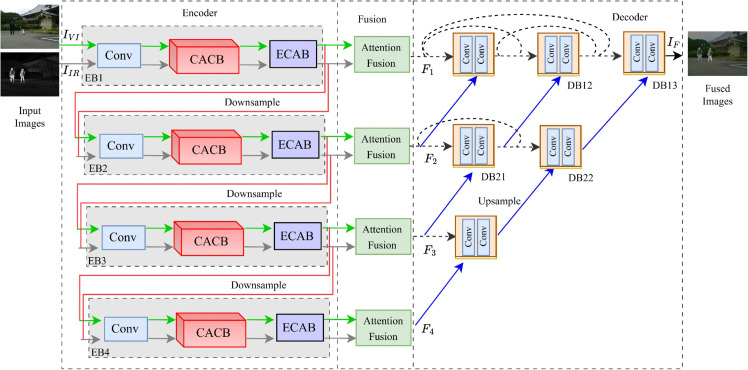
Schematic diagram of the proposed CAFusion. The framework consists of an encoder with four streams for multi-level modality-specific feature extraction, followed by a fusion layer and a multiscale nested decoder for fused image reconstruction.

To address these limitations, we propose CAFusion, a progressive IR and VI fusion network that combines a context-aware convmixer and multi-level fusion to retain global context and modality-specific information with low computational cost. This design enhances its suitability for practical applications while addressing the limitations of current approaches. Different from the single hierarchical flow of the network, we introduce a multilevel progressive feature extraction scheme to capture multiscale features. This scheme enables the encoding network to keep local and global features at multiple scales. In particular, the context-aware convmixer expands the receptive field via dilated and depthwise separable convolutions to capture local and long-range cues efficiently, avoiding the heavy computation of transformer-based modules. As shown in Fig 1, our proposed network consists of multiple streams for IR and VI images. Each stream consists of convolutional, context-aware convmixer blocks and efficient channel attention blocks designed to extract modality-specific features. From top to bottom, each stream process progressively downsamples inputs, allowing the network to mine features at various scales and depths. The network extracts and fuses multiscale features at each level to create intermediate feature embeddings. These are then processed by the subsequent branch of the nested decoder to produce the fused image. In short, the main contributions of this study can be summarized as:

- We introduce CAFusion, a progressive multiscale fusion network for IR and VI images that preserves both local textures and global context through stepwise integration at multiple scales.
- We design a context-aware convmixer block that combines dilated and depthwise separable convolutions to efficiently extract modality-specific and contextual features, while significantly reducing the computations compared to standard convolutional layers.
- We propose an attention-guided intermodality fusion strategy that adaptively weights multi-level features, enhancing complementary information exchange without heavy transformer overhead.
- On the TNO benchmark, CAFusion achieves up to 2% higher SSIM than leading transformer-based fusion methods, demonstrating superior fusion quality and efficiency.

## 2 Related work

Due to the strong feature extraction capabilities of multi-layered networks, DL-based frameworks have made significant progress in solving low-level vision problems [20,21]. Recent advancements in DL approaches have drawn the interest of researchers working on IR and VI image fusion. Comparing these methods to conventional algorithms, most of them have shown state-of-the-art performance utilizing unsupervised learning techniques [22,23]. This section briefly describes the most representative DL-based image fusion models.

### 2.1 Learning-based fusion techniques

Initially, a siamese CNN architecture as a part of the whole algorithm was proposed to fuse IR and VI images. Liu et al. [24], designed a CNN-based siamese network for IR and VI image fusion. This method utilizes CNN for patch classification to generate a weight map, followed by image processing techniques to produce fused images. However, due to the lack of ground truth data, autoencoder (AE)-based unsupervised learning schemes have become popular in the multisensor image fusion field. AE-based approaches utilize encoders for feature extraction and a fusion layer to fuse image features and a decoder to reconstruct the fused result. Li et al. [10] introduced an unsupervised fusion approach that utilizes densely connected layers to extract features, fuse them, and subsequently reconstruct them by employing a decoder.

Along with AE-based methods, utilizing an adversarial game between a generator and a discriminator, generative adversarial network (GAN)-based techniques are introduced to fuse VI and IR images. FusionGAN [14] was the first model to employ GAN for IR and VI fusion. A discriminator was developed to separate the fused image from the VI image, forcing the generator to keep the IR and VI image’s saliency details and the feature information, respectively. The method presented by Gao et al. [25] is based on the densely connected disentangled representation GAN, which separates and combines the modal details and content of VI and IR images using a disentangled representation.

Recently, due to long-range modeling capabilities, self-attention and vision transformer (ViT) based methods are gaining popularity in image fusion applications. These techniques introduced attention or ViT in the AE and GAN baseline architectures. To efficiently extract significant details from multimodal images, Rao et al. [26] proposed AT-GAN, a GAN-based technique integrating intensity attention and semantic transition modules. Tang et al. [16] introduced a ViT-based technique to acquire local and global features of source images. Ma et al. [17] presented SwinFusion for image fusion that makes use of Swin Transformer and cross-domain long-range learning. The technique combines a shifting windows mechanism to handle images of any size with attention-guided cross-domain modules for efficient information integration and global interaction.

Recently, some advanced fusion techniques have emerged. T2EA [27] introduces a target-aware Taylor Expansion Approximation Network that explicitly enhances salient target representation. DSAFusion [28] employs a detail-semantic-aware architecture tailored for IR and low-light VI fusion to jointly preserve fine textures and semantics. MSCS [29] conducts multi-stage feature learning with channel–spatial attention for fuison, leveraging attention to capture global saliency and texture. MSPIF [30] performs multi-stage progressive fusion with structure preservation to maintain edges and contours across scales. HaIVFusion [31] proposes a haze-aware IR–VI fusion framework that jointly suppresses haze effects to recover richer visible textures while preserving salient IR targets.

Many existing image fusion methods suffer a trade-off between fusion quality and computational complexity. Traditional deep learning models often rely on complex convolutional networks that capture local features well but struggle with long-range dependencies, causing loss of global contextual information. On the other hand, transformer-based methods have demonstrated superior performance by effectively modeling long-range interactions through self-attention mechanisms. Despite this, several challenges remain, such as attention mechanisms often only capturing common features without effectively separating discrepancy information between modalities. Additionally, due to the quadratic complexity of self-attention, many ViT-based methods use fixed patch sizes or simplistic attention schemes, restricting their ability to balance global context with fine local details. Motivated by these limitations, we introduce a convmixer-based alternative, which replaces attention with a combination of dilated and depthwise separable convolutions. This design retains the capacity to capture both local and global context efficiently, drastically reducing complexity while maintaining or improving fusion performance.

## 3 Method

### 3.1 Network overview

The fusion framework first learns feature extraction at multiple levels for the given source images $I_{VI}\in {\mathbb{R}}^{C\times H\times W}$ and $I_{IR}\in {\mathbb{R}}^{C\times H\times W}$ where *C*, *H*, and *W* indicate the number of channels, height, and width, respectively. Next, the features of $I_{VI}$ and *I*_*IR*_ at each level are combined to form feature embeddings. Each of these embeddings is then passed through the nested decoder at its corresponding level to produce the fused image $I_{F}\in {\mathbb{R}}^{C\times H\times W}$. The overall framework consists of three modules, as shown in Fig 1. Initially, conv and context-aware convmixer blocks are utilized for multiscale feature extraction. To efficiently extract local and complementary information from both modalities, the fusion block is utilized to combine the complementary and modality-specific features at each scale. The final fused image is then reconstructed using the network’s decoder, which operates on nested connections.

### 3.2 Feature extraction

**Fig 2:**
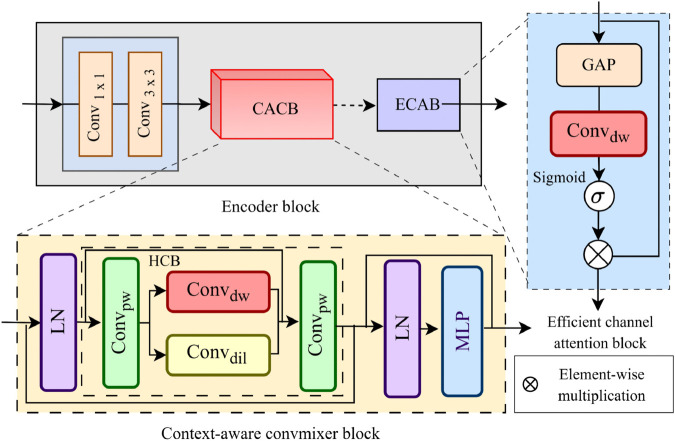
Architecture of the proposed encoder block, which further consists of a conv block, a context-aware convmixer block and an efficient channel attention block.

The proposed encoder consists of several encoder blocks (EB), each utilizing conv, context-aware convmixer blocks (CACB) and Efficient channel block (ECAB). The architecture of an EB at level *l* is shown in Fig 2. Initially, the conv block is employed for the extraction of shallow features of $I_{VI}$ and *I*_*IR*_ and the downsampling of existing features. The conv block can efficiently extract local semantic details while downsampling the input features. Conv block consists of two sequential conv layers. The first conv layer performs a 1×1 convolution for extracting shallow features. The second layer employs a convolution with a stride *s* to achieve down-sampling. The whole operation on the conv block can be expressed mathematically as:

$$F_{IR/VI}^{l}={Conv}_{s}({Conv}_{1}(IR/VI))$$ (1)

where, $I_{IR/VI}$ is the input to conv block, *l* represents the level of the encoder block. $Conv_{1}$ is the 1x1 conv operation for shallow feature extraction and $Conv_{s}$ is the conv operation with a stride *s* for the downsampling process. The output of the conv block is further processed in the subsequent level with the same conv block for a further downsampling of the input of the preceding level. Instead of using the standard max pooling, we chose strided convolution, which is more efficient while preserving important information. Unlike max pooling, strided convolution allows us to reduce the size of the image while retaining important detail and improving features. Furthermore, strided convolution facilitates smooth information flow, allowing our model to learn from the whole image without missing any crucial details.

#### 3.2.1 Context-aware convmixer block.

This section explains the proposed Context-Aware Convmixer block (CACB). Unlike standard convmixer architectures [32] that rely primarily on sequential depthwise separable (DS) and pointwise (Pw) Convs for spatial and channel mixing, our CACB introduces a hybrid parallel approach. Traditional convmixers typically expand receptive fields through larger kernels or deeper stacking, which can be computationally inefficient. In contrast, CACB employs two parallel convolutional pathways: DSConv for efficient local feature extraction and dilated convolutions (DilConv) for capturing long-range dependencies without parameter overhead. This design enables simultaneous processing of fine-grained spatial details and broader contextual information, making it particularly suitable for multi-modal image fusion, where both local texture preservation and global structure understanding are critical. The architecture of the proposed CACB is presented in Fig 2. Given the input features $F_{IR/VI}^{l}$ at level *l* first, the layer normalization (LN) is applied to speed up and stabilize training to get normalized features as:

$$F_{IR/VI}^{l^{\prime }}=L_{N}(F^{l}I_{IR/VI})$$ (2)

The normalized features are then processed through a Hybrid Convolutional Block (HCB), which consists of different conv layers to independently extract spatial and contextual local and global features before being merged. The refined output undergoes a second layer normalization and is subsequently processed through a multilayer perceptron (MLP) to produce the final feature representation. Within the HCB, the normalized input features are first passed through an initial pointwise convolution (PwConv) to enhance inter-channel interaction. A DSConv is employed that enhances spatial feature extraction while maintaining computational efficiency. By separating spatial and channel processing, DSConv allows the model to capture fine details and complex dependencies. In parallel, a DilConv layer is utilized to expand the receptive field, capturing long-range dependencies and improving multi-scale feature extraction without increasing the parameter count. The features from DSConv and DilConv are passed through a second PwConv to refine the extracted features before merging them with a residual connection to retain the original information. The HCB can be formulated as follows:

$$HCB(F_{IR/VI}^{l^{\prime }})=C_{pw2}\left(C_{ds}(C_{pw1}(F_{IR/VI}^{l^{\prime }})+C_{dil}(C_{pw1}(F_{IR/VI}^{l^{\prime }}))\right)+F_{IR/VI}^{l^{\prime }}$$ (3)

where $HCB(F_{IR/VI}^{l^{\prime }})$ represents the output of HCB and *C*_*pw*1_, *C*_*pw*2_, *C*_*ds*_ and *C*_*dil*_ denote initial PwConv, second PwConv, DSConv and DilConv respectively. The dilation rate of dilConv is set to 2 in each HCB block. By combining the $Conv_{ds}$ and $Conv_{dil}$ features, CACB improves both localized spatial structures and global context at the same time. This makes features more expressive while keeping the computational load low. Following the HCB, the output features undergo another LN to further refine the representation. An MLP consisting of two fully connected layers with ReLU activation is then applied, enhancing high-level feature abstraction. Mathematically, the final formulation of the CACB is:

$$Y_{IR/VI}^{L}=HCB(F_{IR/VI}^{l^{\prime }})+F_{IR/VI}^{l}+MLP\left(L_{N}\left(HCB(F_{IR/VI}^{l^{\prime }})\right)\right)$$ (4)

#### 3.2.2 Efficient channel attention block.

To further enhance the deep feature representation of EB, we introduce the Efficient Channel Attention Block (ECAB). The architecture of the ECB is presented in Fig 2 (right). Given an input feature map $F_{IR/VI}^{l}$, ECAB adaptively recalibrates channel-wise features through a lightweight attention mechanism. The structure of ECAB is a step-by-step process in which the input features go through global average pooling (GAP) to combine spatial information and make a channel descriptor. Then a lightweight 1×1 depthwise convolution is applied to capture inter-channel dependencies efficiently. The transformed features are passed through a sigmoid function to generate channel-wise attention weights. Finally, the original input features $F_{IR/VI}^{l}$ to ECAB are rescaled by the computed attention weights via element-wise multiplication to produce the refined output $F_{IR/VI}^{l^{\prime }}$. Mathematically, ECAB steps can be formulated as:

$$F_{IR/VI}^{l^{\prime }}=\sigma (Conv_{dw}^{1\times 1}(GAP(F_{IR/VI}^{l})))⊙F_{IR/VI}^{l}$$ (5)

where σ(·) represents the sigmoid activation function, and ⊙ denotes element-wise multiplication. The output features of ECAB from each IR and VI stream are utilized for next-level feature extraction and feature fusion in the next stage.

### 3.3 Feature fusion

We propose a fusion strategy integrating spatial attention-based weighting for multi-scale deep feature fusion. Given two input feature maps $F_{VI}^{l}$ and $F_{IR}^{l}$ extracted from an EB at level *l*. We apply a spatial attention mechanism to determine the relative importance of each modality at every spatial location. The spatial importance of each modality is computed using L1-norm pooling. The spatial weight maps $W_{VI}^{l}$ and $W_{VI}^{l}$ are defined as:

$$W_{IR}^{l}(x,y)=\frac{|F_{IR}^{l}(x,y)|1}{|F_{IR}^{l}(x,y)|1+|F_{VI}^{l}(x,y){|}_{1}}$$ (6)

$$W_{VI}^{l}(x,y)=\frac{|F_{VI}^{l}(x,y)|1}{|F_{IR}^{l}(x,y)|1+|F_{VI}^{l}(x,y){|}_{1}}$$ (7)

These weights determine the contribution of each modality to the fused feature map. The weighted features are computed as:

$$F_{IR}^{l^{\prime }}(x,y)=W_{IR}^{l}(x,y)\cdot F_{IR}^{l}(x,y)$$ (8)

$$F_{VI}^{l^{\prime }}(x,y)=W_{VI}^{l}(x,y)\cdot F_{VI}^{l}(x,y)$$ (9)

Finally, the fusion process applies a mean operation to obtain the final fused feature map:

$$F_{F}^{l}=\frac{F_{IR}^{l^{\prime }}+F_{VI}^{l^{\prime }}}{2}$$ (10)

This ensures a balanced fusion of IR and VI features while maintaining the structural integrity of both modalities. We choose the L1-norm for computing spatial importance due to its ability to measure the overall magnitude of feature activations at each spatial location robustly and efficiently. Unlike the L2-norm, which disproportionately emphasizes large activation values, or max-pooling that only captures the strongest response, the L1-norm aggregates information evenly across all channels. This property ensures that the spatial attention weights reflect a comprehensive and balanced importance measure of the input features, leading to stable fusion weights that preserve complementary information from both IR and VI modalities.

### 3.4 Reconstruction

The decoder network employs a U-Net++ architecture with nested connections for effective information integration, as illustrated in Fig 1. The decoder consists of six blocks denoted as *DBln*, where *DB* denotes the decoder block, *l* is the depth level and *n* represents the block number. These blocks are responsible for processing the fused features at different levels. For instance, DB11 takes the fused features from the first level with upsampled features from the second level, while D21 handles fused features from the second level alongside upsampled features from the third level, and so on. This hierarchical arrangement of decoder blocks facilitates the progressive reconstruction of features, capturing both local and global contextual information from multiple scales. This design avoids cumulative information loss and suppresses noise amplification by continuously reinforcing early-stage details throughout the fusion and reconstruction process.

### 3.5 Training

During the training phase, an auto-encoder network is trained where the encoder is proficient in extracting multi-scale deep features, and the fused image is generated by the decoder based on these extracted features. To train the autoencoder network, we employed a comprehensive loss function to guide the optimization process. The loss function is composed of three distinctive components: SSIM loss to preserve structural similarity and maintain overall perceptual quality, pixel-wise loss to minimize pixel-wise differences, ensuring accurate reconstruction, and gradient loss to retain fine texture details critical for edge preservation, for better fusion results. The SSIM loss is formulated based on the differences between the input images and their corresponding fused output, which can be defined as:

$${ℒ}_{\text{ssim}}=1-\text{SSIM}(I_{F},I)$$ (11)

where SSIM can be computed as:

$$\text{SSIM}(I,I_{F})=\frac{\left(2\mu_{I}\mu_{I_{F}}+C_{1})\cdot (2\sigma_{II_{F}}+C_{2}\right)}{\left(\mu_{I}^{2}+\mu_{I_{F}}^{2}+C_{1})\cdot (\sigma_{I}^{2}+\sigma_{I_{F}}^{2}+C_{2}\right)}$$ (12)

where, I and *I*_*F*_ are the local patches of input and output images, $\mu_{I}$ and $\mu_{I_{F}}$ represents the average intensities. The pixel-wise loss (${ℒ}_{\text{pixel}}$) measures the absolute difference between the input image *I*_*ij*_ and the reconstructed output image *I*_*Fij*_ normalized by the total number of pixels *np* as:

$${ℒ}_{\text{pixel}}=\frac{1}{\text{np}}\sum_{i=1}^{\text{np}}|I_{ij}-I_{Fij}|$$ (13)

This loss measures how well the pixel values in the reconstructed image match those in the original, considering variations in brightness. Finally, the texture loss evaluates the differences in image gradients between the combined image and the individual input images. It employs Sobel operators to calculate image gradients, providing insights into the image’s local structures.

$${ℒ}_{\text{text}}=\frac{1}{H\times W}{\left\|\left|\nabla (I_{F})-\text{avg}(\left|\nabla (I)\right|)\right|\right\|}_{1}$$ (14)

where ∇ is the Sobel gradient operator, assessing the texture details within an image. |.| represents the absolute operation, ||.||_1_ denotes the l1-norm, and avg(.) signifies the element-wise average selection. Finally, the comprehensive objective function for our fusion model is expressed as a weighted sum of all sub-loss terms:

$${ℒ}_{\text{total}}=\alpha {ℒ}_{\text{ssim}}+\beta {ℒ}_{\text{pixel}}+\gamma {ℒ}_{\text{text}}$$ (15)

where α, β, and γ denote the respective weights assigned to the structural similarity loss, pixel-wise loss, and texture loss which also act as balancing parameters. This study sets the values of α, β, and γ as 1.0, 0.01 and 0.1 respectively.

## 4 Experimental results and discussion

In this section, we provide a comprehensive overview of the experiment setup and results for the proposed fusion method. Then we present a comparative analysis between the proposed fusion approach and various other approaches, employing both qualitative and quantitative evaluations. In the ablation study section, we investigate the impact of variations in the network structure. Finally, we provide a comprehensive discussion and analysis of the overall results obtained from our experiments.

### 4.1 Implementation details

The encoders follow a four-level hierarchical design with channels progressively set as [16, 32, 64, 128]. Each level employs conv blocks for down-sampling, using stride-based convolutions. PwConvs and DSconvs in the CACB utilize 1×1 and 3×3 kernel sizes, respectively and DilConv uses a 3×3 kernel with a dilation rate set as 2. The decoder is implemented as a nested structure with bilinear upsampling for feature alignment. The final output is produced via a 1×1 convolution layer that maps the fused feature maps into a single-channel fused image. We collected 2000 infrared and visible image pairs from the FLIR-roadscene dataset and extracted 64×64 patches from input image pairs for training. The model is optimized using the Adam algorithm with an initial learning rate of 1 $\;\times \;10^{-4}$ and momentum parameters β=(0.9,0.999). Learning rate scheduling employs MultiStepLR with milestones at $\frac{2}{3}$ and $\frac{8}{9}$ of the total epochs, decaying the rate by a factor of 0.1 at each milestone. Training proceeds for 300 epochs, with convergence determined by monitoring the validation loss early stopping is triggered if the loss does not improve by at least 0.001 over 10 consecutive epochs, and the best model is selected based on the lowest validation loss. The whole framework is implemented using the PyTorch library, and the model training process is executed on a workstation with having NVIDIA RTX 3090 GPU.

For RGB VI image fusion we utilize the commonly used approach [33] to incorporate the color information in the final result. First, the source VI images are transformed into the YCbCr domain. Then the Y channel of the VI image is fused with the IR image to get the gray fused result. Finally, the RGB fused image is obtained by merging the Cb and Cr channels of the VI image with the gray fused images. This strategy is employed for roadscene and MSRS datasets. In this study, we chose nine fusion methods to compare with the proposed method for subjective and objective evaluation. These techniques include DenseFuse [10], CNN-based unified fusion network (IFCNN) [34], NestFuse [11], TarDAL [1], SeAFusion [35], SwinFusion [17], IGNet [36], DDFM [19] and CDDFuse [37]. We utilize three datasets, including TNO [38], FLIR-roadscene [39], and Multi-Spectral Road Scenarios (MSRS) [40,41] for comparative evaluation.

### 4.2 Subjective evaluation

**Fig 3:**
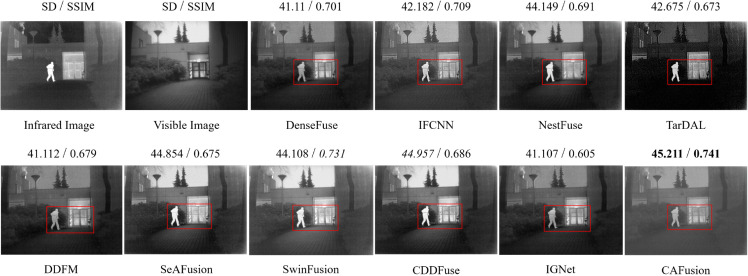
Fused results of the Kaptein1123 image pair from the TNO dataset. Each fused image is annotated with its standard deviation and SSIM score. The bold values indicate the best performance, while italic values denote the second-best result.

Image fusion aims to generate images with improved additional details for human vision. However, there is no benchmark criterion for assessing subjective quality. The assessment considers visual data subjectively from three origins: the fused image, the two separate images, and human perception. All three elements can indicate the quality of the fused image [42]. We examine the DL-based SOTA and recent methods to validate the effectiveness of our proposed model. To improve the objectivity of our qualitative evaluation, we also included additional quantitative metrics (standard deviation and SSIM) for each fusion result. Fig 3 shows the fused results from the TNO dataset Kaptein1123 image pair and compared with the proposed CAFusion.

To give an intuition of more specific comparisons, we mark the infrared object and the door in the scene with the red rectangle. That allows us to assess both the clarity of targets and edge details preservation. The NestFuse method cannot preserve the floor texture, resulting in more infrared modality details in the fused result. SeAFusion and SwinFusion merge more visible details while failing to balance visible and infrared information. whereas, TarDAL, DFFM and IGNet overemphasize infrared information and generate dark images with few visible details, affecting the scene’s comprehension. Moreover, the edges are not reconstructed properly in the TarDAL method, which introduces blurry artifacts in the whole image. Our proposed CAFusion uniformly fuses the information from both VI and IR modalities. This allows infrared objects and key details, like the door, to be sharp and well-defined in the fused image.

**Fig 4:**
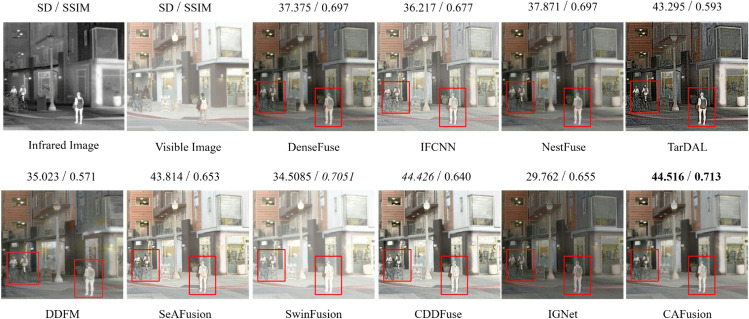
Visual fused results of the sample pair from the road scene dataset. SD and SSIM scores are presented with each result. The best and second-best values are marked in bold and italic, respectively.

**Fig 5:**
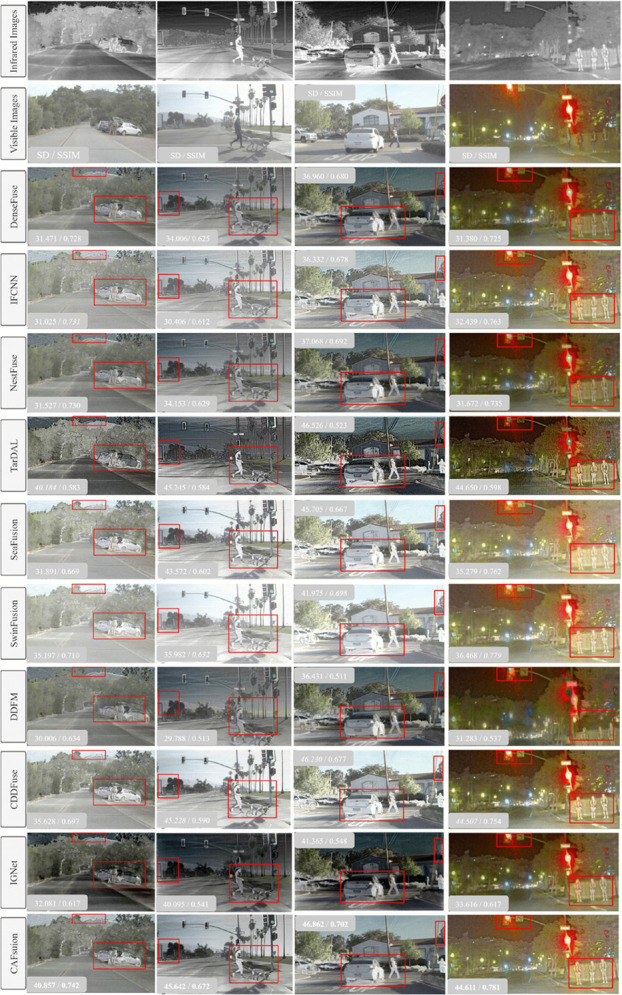
Fused results of four representative image pairs from the RoadScene dataset. Quantitative results are presented alongside each fused image, reporting SD and SSIM values. Bold and italic highlights are used to mark the best and second-best scores, respectively.

For a comprehensive evaluation of the visual fusion results, we presented the fused images from the roadscene dataset in Figs 4 and 5. To provide an understanding of the comparisons, we highlight the IR objects, such as pedestrians and some objects in the scene, with a red rectangle. The results obtained through the IFCNN, SeAFusion, SwinFusion and CDDFuse methods exhibit noticeable VI modality details and appear more bright and comparable to the VI image with comparable SSIM and SD scores. Although these methods incorporate infrared information, some small details are missing from the fused results due to brightness. TarDAL and IGNet techniques produce darker images while focusing more on IR modality details, overachieving balanced information between IR and VI modalities, therefore achieving the lowest SSIM scores. The DDFM method failed in the reconstruction of edges, such as bicycles are not properly reconstructed in Fig 4. The output generated by the proposed CAFusion is better than the other techniques, where the reconstructed objects are much sharper. That proves its efficiency in preserving modality-specific details by keeping the proper balance of the fusion between VI and IR information. This demonstrates the effectiveness of our proposed CAFusion in achieving superior fusion results while maintaining visual quality and modal balance.

**Fig 6:**
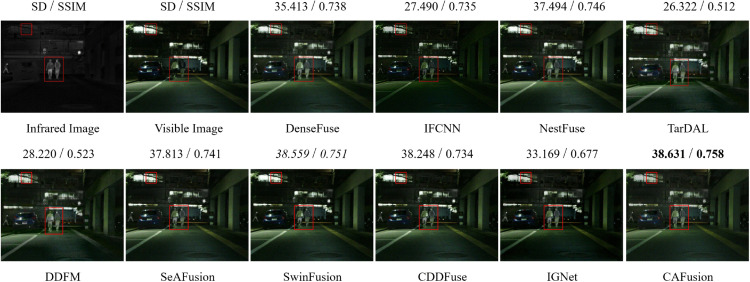
Fused results of a low-light sample pair from the MSRS dataset. For each method, SD and SSIM scores are shown on the corresponding fused image. Bold values represent the best-performing method, while italic values mark the second-best.

For more visual comparison, Fig 6 shows fused results from the 01348N image pair in the MSRS dataset. This pair represents a challenging nighttime scene with low lighting. To highlight differences, pedestrians in the IR image are marked with red rectangles, while small red boxes indicate window edges for evaluating edge preservation. Although the fused results appear similar, quantitative metrics provide deeper insights. Methods like TarDAL and DDFM struggle to retain structural details, reflected in their low SD and SSIM scores. CDDFuse and SwinFusion perform better, with SwinFusion achieving the second-best scores after the proposed CAFusion. Our CAFusion produces a balanced fusion—clear and bright images with preserved edges and enhanced object visibility, which is vital for detection tasks.

### 4.3 Quantitative evaluation

The fused results are also evaluated objectively using objective evaluation fusion metrics, however, there is no benchmark for selecting fusion metrics for evaluation. In this study, five objective assessment metrics are utilized to compare our method with SOTA and recent DL methods to make a fair and thorough comparison. These metrics include Entropy (*EN*) [43], standard deviation (*SD*) [44], image structural similarity (*SSIM*) [45], gradient-based metric (*Q*_*ab*/*f*_) [46] and visual information fidelity (*VIF*) [47]. The fusion algorithm’s performance gets better when these metrics’ total score increases. Higher values of *EN* show that the fusion method retains more amount of information from the source images. *SD* evaluates the contrast and distribution of pixel intensities. High values of *SSIM* and *Q*_*ab*/*f*_ indicate that the fusion algorithms preserve more structural and edge information from the source images, respectively. Whereas, *VIF* evaluates the fidelity of the fused image as seen by the human visual system. Tables 1, 2 and 3 provides a comprehensive quantitative evaluation of various image fusion techniques across three datasets: TNO, Roadscene, and MSRS respectively, using five fusion metrics. We report both the average and standard deviation of each metric; including these statistics enables a more accurate evaluation of the methods’ consistency and the statistical significance of the improvements. The best and second-best values are highlighted in bold and italicized respectively. The results for the TNO dataset are based on the average scores of 23 image pairs. The Roadscene dataset and the MSRS dataset are evaluated using 50 test image pairs.

**Table 1:** Quantitative comparison of fused images from the TNO dataset.

Method	*EN*	*SD*	*SSIM*	$Q_{ab/f}$	*VIF*
DenseFuse	6.752 ± 0.215	32.68 ± 1.625	0.731 ± 0.115	0.451 ± *0.039*	0.627 ± 0.122
IFCNN	6.595 ± 0.195	32.36 ± 1.890	0.735 ± *0.113*	0.441 ± 0.047	0.619 ± 0.123
NestFuse	6.892 ± 0.175	41.637± 1.584	0.749 ± 0.145	0.453 ± 0.069	0.749 ± 0.158
TarDAL	7.067 ± 0.243	*43.708* ± 1.981	0.695 ± 0.149	0.412 ± 0.052	0.643 ± 0.119
SeAFusion	7.127 ± 0.197	43.376 ± 1.631	0.736 ± 0.116	0.455 ± 0.059	0.697 ± 0.145
SwinFusion	6.985± 0.304	41.059 ± 1.765	0.731 ± 0.145	0.459 ± 0.062	*0.756* ± 0.145
DDFM	7.063 ± 0.281	41.529 ± 1.540	0.682 ± 0.138	0.413 ± 0.150	0.689 ± 0.299
CDDFuse	*7.121* ± 0.254	41.653 ± 1.792	*0.755* ± 0.136	0.459 ± 0.068	0.754 ± *0.177*
IGNet	7.091 ± *0.146*	40.381 ± *1.384*	0.730 ± 0.147	*0.461* ± 0.058	0.741 ± 0.118
CAFusion	**7.153± 0.139**	**43.961 ± 1.269**	**0.769 ± 0.091**	**0.469 ± 0.035**	**0.769 ± 0.120**

**Table 2:** Quantitative results of fused images from roadscene dataset.

Method	*EN*	*SD*	*SSIM*	$Q_{ab/f}$	*VIF*
DenseFuse	6.671 ± 0.161	30.682 ± 1.191	0.719 ± 0.085	0.401 ± *0.045*	0.668 ± 0.113
IFCNN	6.768 ± 0.149	31.161 ± 1.596	0.716 ± 0.105	0.398 ± 0.057	0.634 ± 0.113
NestFuse	7.312 ± 0.160	*46.137*± 1.184	0.739 ± 0.134	0.468 ± 0.038	0.739 ± 0.140
TarDAL	7.219 ± 0.163	45.078 ± 1.036	0.684 ± 0.093	0.435 ± 0.042	0.596 ± 0.115
SeAFusion	7.238 ± 0.120	43.332 ± 1.531	0.729 ± *0.050*	0.468 ± 0.040	0.721 ± 0.134
SwinFusion	**7.348**± 0.191	42.059 ± 1.681	0.731 ± 0.069	*0.482* ± 0.052	*0.761* ± 0.122
DDFM	7.165 ± 0.155	41.529 ± 2.125	0.705 ± 0.113	0.419 ± 0.041	0.705 ± *0.088*
CDDFuse	*7.335* ± 0.105	45.531 ± 8.792	*0.737* ± 0.066	0.481 ± 0.041	0.781 ± 0.145
IGNet	7.062 ± *0.059*	45.381 ± *1.025*	0.718 ± 0.090	0.473 ± 0.038	0.674 ± 0.123
CAFusion	7.332 ± **0.051**	**46.396 ± 1.019**	**0.748 ± 0.045**	**0.511 ± 0.034**	**0.791 ± 0.075**

**Table 3:** Quantitative comparison of fused images from the MSRS dataset (mean ± standard deviation). The best and second-best results for each metric are highlighted in bold and italicized, respectively.

Method	EN	SD	SSIM	$Q_{ab/f}$	VIF
DenseFuse	5.961 ± 0.243	33.223 ± *1.245*	0.693 ± 0.067	0.408 ± 0.065	0.659 ± 0.118
IFCNN	5.854 ± 0.220	33.109 ± 1.439	0.691 ± 0.070	0.420 ± 0.065	0.635 ± 0.116
NestFuse	6.171 ± 0.250	39.622 ± 1.968	0.734 ± 0.071	0.432 ± *0.055*	0.731 ± 0.143
TarDAL	6.151 ± 0.161	36.705 ± 1.395	0.692 ± 0.071	0.407 ± 0.059	0.653 ± *0.115*
SeAFusion	*6.803* ± 0.125	38.690 ± 1.929	0.728 ± 0.070	0.449 ± 0.086	0.734 ± 0.134
SwinFusion	6.290 ± 0.129	41.302 ± 1.981	*0.751* ± 0.060	0.467 ± 0.085	0.755 ± 0.134
DDFM	6.599 ± 0.148	40.264 ± 1.986	0.718 ± 0.055	0.419 ± 0.065	0.616 ± 0.126
CDDFuse	6.743 ± *0.113*	**43.184** ± 1.885	0.743 ± *0.052*	*0.474* ± 0.095	*0.761* ± 0.164
IGNet	6.210 ± 0.110	40.506 ± 1.299	0.722 ± 0.055	0.454 ± 0.076	0.613 ± 0.123
CAFusion	**6.866** ± **0.106**	*42.812* ± **1.237**	**0.756** ± **0.050**	**0.484** ± **0.051**	**0.772** ± **0.112**

Table 1 shows that DDFM and TarDAL perform weakly in maintaining detailed texture information, leading to lower *EN* and *SD* scores in the TNO dataset. This results in a lack of detail and contrast in the fused images. Although SeAFusion and SwinFusion perform well in terms of *SSIM*, their moderate scores in *Q*_*ab*/*f*_ and *VIF* indicate that they fail to maintain a decent balance between the preservation of VI information and IR features. As a result, the images appear brighter and more visually saturated. NestFuse and CDDFuse achieve strong results in *EN* and *SSIM* respectively, indicates that these methods retain significant information and preserve structural features well. SeAFusion also demonstrates good *EN* and *SSIM*, but its performance in *Q*_*ab*/*f*_ and *VIF* is slightly weaker. On the other hand, our proposed technique, CAFusion, outperforms all of the metrics in the TNO dataset. This demonstrates that our approach is more capable of preserving image detail and contrast. Higher *SSIM* and *Q*_*ab*/*f*_ values indicate very good structural and edge preservation.

DDFM and TarDAL failed to preserve image contrast and edge information for the Roadscene dataset in Table 2. IFCNN, in particular, misses out on small details, as seen by its relatively low *EN* and *SD* values. While TarDAL may maintain a higher *EN* score, it produces significantly darker images with less visible detail, resulting in poor scores for *Q*_*ab*/*f*_ and *VIF*. NestFuse has higher values for *EN*, indicating that it retains significant information with high visual information fidelity. Similarly, SwinFusion achieves the better *SSIM* score, indicating it preserves structural information very well. However, average scores in metrics such as *EN*, *VIF*, and *Q*_*ab*/*f*_ result in fused images that are brighter and looks closer to the visible modality. Our proposed CAFusion approach has the best performance, achieving the best scores in 4 metrics, displaying strength in structure preservation with clear edges and retaining a high level of visual fidelity.

Most methods, such as IFCNN and IGNet, show either information retention or edge preservation problems in the MSRS dataset in Table 3. IFCNN has a lower score for *EN* and *SSIM*, resulting in fused images with less information retention. Although IGNet performs reasonably in *VIF*, the texture details are poorly preserved. CDDFuse performs better in preserving image structural and edge information of the image compared to the other techniques. The second-best value of *VIF* indicates that the Swinfusion method retains high visual information fidelity. CAFusion possesses an overall top performance across all metrics. That reflects the performance of CAFusion in generating high-quality fused images characterized by a good trade-off between information retention, structural preservation, and visual fidelity. Although our algorithm generally outperforms the others, slightly lower scores in certain metrics, like *EN* or *SD*, may be due to the specific diversity of the dataset images.

### 4.4 Ablation study and discussion

We conducted ablation studies to analyze the variations and details of the proposed CAFusion framework. We trained and tested various network choices and provided a comprehensive analysis.

#### 4.4.1 Effect of CACB and ECAB.

In this section, we conduct a series of ablation experiments to analyze the contributions of the proposed Context-Aware Convmixer Block (CACB) and Efficient Channel Attention Block (ECAB) to the fusion framework. In this evaluation, we compare the performance of the model with and without CACB as well as the combination of CACB with ECAB in the encoder blocks (EB). We tested different architectural configurations of EBs that include *B*: baseline architecture that consists of EBs with convolutional blocks with no CACB and ECAB. In the second configuration *C*: CACB is incorporated to the EBs without dilated convolution in the CACB to assess its effectiveness in enhancing feature extraction. Next configuration *C*2: integrates dilated conv in CACB (proposed CACB). Final configuration *C*3: includes ECAB with CACB in the EBs.

Table 4 presents the results of tested configurations evaluated on five widely used fusion metrics on the TNO dataset. From the table we can see that CACB without dilation (C1) marginally improves structural metrics like *SSIM*. Adding dilated convolutions (C2) significantly enhances the receptive field, leading to improvements in *EN*, *SSIM*, and *VIF*. Combining CACB with ECAB (C3) further boosts performance across all metrics, confirming the complementary benefits of contextual mixing and channel-wise attention. The results confirm that the best performance is achieved in C3, validating the combination of CACB and ECAB for improved feature extraction.

**Table 4:** Effect of CACB, Dilated Convolutions, and ECAB Integration.

Configuration	*EN*	*SD*	*SSIM*	$Q_{ab/f}$	*VIF*
B	6.863	41.602	0.748	0.458	0.745
*C*1	6.531	41.103	0.751	0.449	0.732
*C*2	*6.985*	*41.059*	*0.751*	*0.459*	*0.753*
*C*3	**7.153**	**43.961**	**0.769**	**0.467**	**0.769**

#### 4.4.2 Effect of CACB integration across encoder levels.

In this experiment, we investigate the impact of CACB by progressively incorporating them into different levels of the encoder blocks (EB). The encoder comprises four hierarchical levels, denoted as *l*_1_ to *l*_4_. We evaluate four different configurations by varying the number and placement of CACBs at each level. Specifically, configuration P1 includes one CACB at each level. In P2, two CACBs are employed at every encoder level. Configuration P3 applies two CACBs at *l*_1_ and *l*_4_ of EBs. Lastly, in P4, levels *l*_1_ and *l*_2_ contain two CACBs, whereas *l*_3_ and *l*_4_ each include one CACB. Five fusion metrics are utilized on the fused images from TNO dataset to validate the performance of each configuration as presented in Table 5.

**Table 5:** Effect of CACB across encoder levels.

Configuration	*EN*	*SD*	*SSIM*	$Q_{ab/f}$	*VIF*
P1	7.018	42.453	0.759	0.462	0.752
P2	*7.149*	*43.959*	0.749	**0.468**	0.765
P3	7.091	43.112	*0.764*	0.465	*0.768*
P4	**7.153**	**43.961**	**0.769**	*0.467*	**0.769**

The quantitative results in the table show that adding more CACBs generally improves fusion performance. Configuration P1, with one CACB at each encoder level, provides better results than the baseline. P2, where two CACBs are added uniformly across all levels, achieves high scores, particularly in *EN*, *SD*, and *Q*_*ab*/*f*_, though it may lead to higher computational cost. The configuration P3, which adds CACBs only at the first (*l*_1_) and last (*l*_4_) levels, shows strong performance in *SSIM* and *VIF*, highlighting the benefit of enhancing both shallow and deep features. P4 (proposed architecture), which uses two CACBs at *l*_1_ and *l*_2_, delivers the overall best scores, especially in *SSIM* and *VIF*, suggesting that focusing additional CACBs at early layers effectively improves texture and edge detail while balancing performance and complexity.

#### 4.4.3 Comparison of different fusion schemes.

To further validate the effectiveness of our fusion approach, we compare CAFusion with alternative fusion strategies, including direct feature addition, concatenation, and attention-based fusion. These fusion techniques are widely used in the image fusion domain, and we evaluate their performance on the TNO dataset. The quantitative results in Table 6 show that our proposed fusion scheme achieves superior results across multiple evaluation metrics, demonstrating its ability to effectively preserve information from both IR and VI modalities.

**Table 6:** Comparison of different fusion schemes.

Method	*EN*	*SD*	*SSIM*	$Q_{ab/f}$	*VIF*
Concatenation	6.932	41.352	0.734	0.445	0.677
Addition	6.453	39.712	0.721	0.438	0.654
Sum Attention Fusion	*6.934*	*42.719*	*0.752*	*0.460*	*0.744*
Mean Attention Fusion (CAFusion)	**7.153**	**43.961**	**0.769**	**0.467**	**0.769**

#### 4.4.4 Effect of loss weights on fusion performance.

Table 7 shows the impact of different weight combinations (α, β, and γ) for SSIM loss, L1 loss, and texture loss on various fusion quality metrics utilizing fused images from the TNO dataset. When the texture loss is zero and the SSIM loss is dominant, the overall quality decreases significantly, indicating insufficient detail preservation. Removing the L1 loss does not affect the performance; however, low scores in the metrics indicate the loss of fine details. While using only the L1 loss and the texture loss model produces the weakest performance, underscoring the essential role of SSIM loss in maintaining structural details. This highlights the importance of the SSIM loss for enhancing the model’s overall performance. A higher L1 loss weight is not beneficial either, as it tends to smooth the image, potentially resulting in the loss of fine details during the reconstruction process. From the table, we can see that when all weights are high (1.0, 1.0, 0.3), the performance is moderate across the metrics. The best results are achieved with balanced weights (0.2, 0.9, 0.1), which improve overall fusion quality. This shows that a proper balance between texture, structural similarity, and pixel-level losses is crucial for producing high-quality fused images.

**Table 7:** Effect of the loss function balancing parameters (α, β, and γ) on the overall fusion quality.

Weights (α, β, γ)	EN	SSIM	SD	VIF	$Q^{ab/f}$
(1.0, 0.1, 0.0)	6.901	0.735	40.815	0.735	0.401
(1.0, 0.0, 0.1)	6.981	0.741	41.293	0.742	0.445
(0.0, 1.0, 0.1)	6.344	0.679	40.071	0.681	0.441
(1.0, 0.1, 0.1)	*7.011*	*0.749*	*41.512*	*0.758*	*0.458*
(1.0, 0.01, 0.1)	**7.153**	**0.770**	**43.914**	**0.769**	**0.467**

#### 4.4.5 Object detection performance

**Fig 7:**
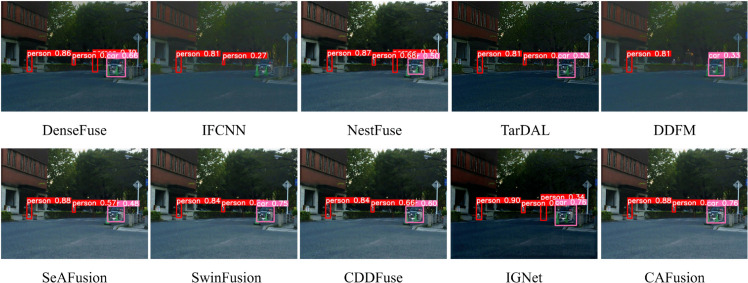
Visualization of object detection results from the 00127D image pair’s fused results within the MSRS dataset.

Object detection is a key task in computer vision that shows how useful fused images are for real applications. We tested all fusion methods using YOLOv5 on 150 images from the MSRS dataset, which has urban road scenes from day and night. The images were labeled with two classes: pedestrians and vehicles. Fig 7 shows detection results on a challenging image (00127D) where pedestrians are hard to see due to low light and dark backgrounds. The visible image alone misses one pedestrian due to poor lighting. Many fusion methods wrongly detect tree stems as pedestrians. DenseFuse, NestFuse, and IGNet show poor object detection with many false positives. DDFM missed detecting one pedestrian, while IFCNN failed to detect the car. SwinFusion and our CAFusion method achieved the best detection accuracy. Table 8 shows the mAP scores for pedestrians, vehicles, and overall detection on IR, visible, and fused images from nine methods. IGNet, CDDFuse, and SwinFusion performed well among existing methods. CAFusion achieves the highest overall mAP score, proving its effectiveness for high-level vision tasks compared to other deep learning fusion methods.

**Table 8:** Comparison of computational cost across transformer-based methods and the proposed ConvFuse.

Method	Pedistrian	Vehichle	mAP
Infrared	0.759	0.764	0.761
Visible	0.512	0.768	0.640
DenseFuse	0.715	0.709	0.712
IFCNN	0.709	0.723	0.716
NestFuse	0.726	0.735	0.730
TarDAL	0.741	0.784	0.762
SeAFusion	0.773	0.781	0.778
SwinFusion	0.779	0.786	*0.782*
DDFM	0.729	0.741	0.735
CDDFuse	*0.789*	0.772	0.780
IGNet	0.767	*0.788*	0.777
CAFusion	**0.805**	**0.792**	**0.798**

#### 4.4.6 Computational cost analysis.

This section analyzes the computational cost of various fusion methods by comparing their average inference time on three benchmark datasets: TNO, RoadScene, and MSRS. The average running time for each method is computed on the same machine, ensuring a fair comparison across different methods. Table 9 presents the average running time (in milliseconds) of each method, where the best values are highlighted in bold. For clarity, we divide the methods based on their underlying architectural complexity: simple convolution-based methods, generative adversarial network (GAN)-based methods, transformer-based methods, diffusion-based methods, and attention-augmented convolutional methods. From the table, we can see that DenseFuse, IFCNN, and NestFuse exhibit the lowest computational cost, as these methods entirely rely on standard convolutional layers for feature extraction. In contrast, methods like SeAFusion, SwinFusion, and CDDFuse incorporate transformers, leading to higher inference times. The DDFM method demonstrates the highest computational cost due to the complexity of its denoising diffusion probabilistic model (DDPM) architecture. On the other hand, our proposed CAFusion achieves competitive computational efficiency, outperforming transformer- and diffusion-based methods while maintaining superior fusion quality. It achieves a favorable balance between performance and computational cost, with inference times comparable to simpler convolution-based methods yet delivering better fusion results.

**Table 9:** Computational cost comparison of different fusion methods across three datasets.

Method	TNO(ms)	RoadScene(ms)	MSRS (ms)
*Convolution-Based Methods*			
DenseFuse	**12.51**	**12.13**	*13.82*
IFCNN	*12.64*	*12.21*	**13.91**
NestFuse	13.23	13.88	15.32
SeAFusion	0.37	0.39	0.38
*GAN-Based Methods*			
TarDAL	15.91	16.35	17.59
*Transformer-Based Methods*			
SwinFusion	40.58	41.09	43.72
CDDFuse	44.09	43.68	45.19
*Diffusion-Based Method*			
DDFM	105K	109K	120K
*Attention-Based Convolutional Methods*			
IGNet	36.29	35.64	40..15
CAFusion	13.39	13.96	15.51

To evaluate the practical efficiency of CAFusion, we measured latency, throughput, and peak memory usage on two hardware configurations: (1) a high-end workstation equipped with an NVIDIA RTX 4090 GPU and an Intel Core i7-14700KF CPU (3.40 GHz), and (2) a low-end desktop with an NVIDIA Quadro P620 GPU and an Intel Core i7-9700 CPU (3.00 GHz). The experiments were performed using images from the TNO dataset with a resolution of 256×256. GPU memory usage was computed using torch.cuda.max_memory_allocated() after clearing the cache, while CPU memory usage was tracked with the psutil library for resident memory and tracemalloc for peak allocations. This ensures that the reported values reflect both the process-wide memory consumption and Python-level peak allocations. Table 10 summarizes the latency, throughput, and peak memory usage.

**Table 10:** Benchmarking results of CAFusion on high-end and low-end devices. Latency is measured in milliseconds per image, throughput in frames per second (FPS), and memory usage in MB. CPU memory is reported as the resident process size.

Device	Peak Memory (MB)	Latency (ms)	Throughput (FPS)
GPU (RTX 4090)	109.16	6.05	165.34
CPU (i7-14700KF)	1040.93	66.68	15.00
GPU (Quadro P620)	89.15	183.67	5.44
CPU (i7-9700)	821.32	259.19	3.86

These results highlight three important observations: (1) GPU acceleration yields significantly lower latency and higher throughput compared to CPU inference, especially on high-end hardware. (2) CPU memory usage is substantially higher than GPU memory usage because resident memory includes the full Python runtime and library overheads, while GPU statistics only capture tensor allocations. (3) On low-end devices, the model remains functional but with reduced throughput (3–5 FPS), illustrating potential trade-offs for real-time deployment. Overall, these experiments demonstrate that CAFusion achieves efficient inference on both high-end and resource-constrained hardware, making it suitable for deployment in real-time multimodal fusion applications.

### 4.5 Discussion

In this study, we proposed CAFusion, a progressive ConvFormer-based network designed for context-aware multisensor image fusion. After extensive evaluations across benchmark datasets, our method demonstrated better performance in terms of quantitative metrics and visual quality. The addition of Context-Aware Convolution Blocks (CACB) and an efficient feature fusion strategy has significantly enhanced the preservation of local-global details from IR and VI modalities. One of the key advantages of our model is its computational efficiency, as compared with complex transformer-based approaches. While CAFusion demonstrates strong performance across various scenarios, several limitations should be acknowledged. The proposed CAFusion method may struggle with extremely low-contrast IR images where thermal information is minimal, potentially leading to over-reliance on VI features and loss of important thermal details. Moreover, severe misalignment between IR and VI images can degrade fusion quality, and the spatial attention mechanism may incorrectly weight features, leading to fusion artifacts and ghosting effects or failure in calibrating IR and VI features in the fused image.

The computational efficiency of CAFusion makes it particularly suitable for several real-world applications where processing speed and resource constraints are critical. In autonomous vehicles, real-time fusion of thermal and visible cameras is essential for nighttime driving and pedestrian detection. For surveillance and security applications, CAFusion’s low memory usage enables potential deployment on edge devices and mobile platforms used in remote monitoring stations, where power consumption and computational resources are limited. Additionally, industrial inspection systems that combine thermal and visible imaging for defect detection can leverage CAFusion’s efficiency for high-throughput quality control in manufacturing environments. While CAfusion is designed for the integration of IR and VI information, its modality-agnostic architecture facilitates its application to other fusion tasks. This is achieved either through custom training on specific datasets or by applying transfer learning techniques. Future work will validate the generalizability by application-specific training such as medical modalities, multi-focus, and multispectral datasets. Overall, CAFusion presents a balanced trade-off between fusion quality, efficiency, and generalizability, making it a promising approach for a wide range of practical applications.

## 5 Conclusion

This paper presented a DL framework for the efficient and effective fusion of infrared and visible images. Our approach addresses the challenges of complex encoding schemes and computational expenses in existing DL algorithms. We introduce a novel multilevel feature extraction mechanism, employing separable and dilated convolutions as an alternative to self-attention within the vision transformer architecture. This modification effectively enhances the extraction of local-global modality-specific features at low cost. Additionally, we proposed a progressive multilevel attention-based fusion strategy, allowing dynamic feature selection from both modalities to enhance fusion quality. Experimental results validate that CAFusion outperforms state-of-the-art fusion methods, including recent transformer-based approaches, in terms of both quantitative metrics and runtime efficiency. In the future, our goal is to improve the CAFusion architecture, explore training strategies on medical image fusion, multi-focus fusion, and multispectral datasets to validate its generalizability across different imaging modalities.
