# Correction: CAFusion: A progressive ConvMixer network for context-aware infrared and visible image fusion

**DOI:** 10.1371/journal.pone.0352432

**Published:** 2026-06-23

**Authors:** Hafiz Tayyab Mustafa, Hamza Mustafa, Hassan Alhuzali, Mujtaba Asad, Zhonglong Zheng

There are errors in the Funding statement. The correct Funding statement is as follows: This research work was funded by Umm Al-Qura University, Saudi Arabia, under grant number: 25UQU4320430GSSR08.

In the Acknowledgement section, the grant number from the funder Umm Al-Qura University, Saudi Arabia is missing. The grant number is: 25UQU4320430GSSR08.
